# The Practice of Midwifery

**Published:** 1899-03

**Authors:** 


**The Practice of Midwifery.** By D. LLOYD ROBERTS, M.D. Fourth Edition. Pp. xv., 585. London: J. & A. Churchill. 1896.

Clear and succinct and quite modern in all its practical part, this book is a very trustworthy manual for students.

The author lays great stress on the proper measures to be used to attain asepsis as far as is possible, and his directions, if followed carefully, will, without being over elaborate, be found amply sufficient.

In describing the mutilating operations for the relief of dystocia he mentions cranioclasm as being the operation of breaking up the base of the skull after craniotomy; we have always been under the impression that this operation was known as basiotripsy while cranioclasm is the operation by which after perforation the vault of the skull is removed piecemeal.

One noteworthy feature is an excellent chapter on symphysiotomy, which gives the completest account of the operation that we have yet met with in any student's manual.

The work, offering a logical and accurate account of the various processes, will form a valuable addition to the student's library. The plates are numerous and well drawn.

